# Ecology: A Prerequisite for Malaria Elimination and Eradication

**DOI:** 10.1371/journal.pmed.1000303

**Published:** 2010-08-03

**Authors:** Heather M. Ferguson, Anna Dornhaus, Arlyne Beeche, Christian Borgemeister, Michael Gottlieb, Mir S. Mulla, John E. Gimnig, Durland Fish, Gerry F. Killeen

**Affiliations:** 1 Biomedical and Environmental Thematic Group, Ifakara Health Institute, Dar es Salaam, United Republic of Tanzania; 2 Faculty of Biomedical and Life Sciences, University of Glasgow, Glasgow, United Kingdom; 3 Department of Ecology and Evolutionary Biology, University of Arizona, Tucson, Arizona, United States of America; 4 International Development Research Centre, Ottawa, Ontario, Canada; 5 International Centre for Insect Physiology and Ecology, Nairobi, Kenya; 6 Foundation of the National Institutes of Health, Bethesda, Maryland, United States of America; 7 University of California, Riverside, California, United States of America; 8 Division of Parasitic Diseases, Centers for Disease Control and Prevention, Chamblee, Georgia, United States of America; 9 Division of Epidemiology of Microbial Diseases, School of Public Health, Yale University, New Haven, Connecticut, United States of America; 10 Vector Group, Liverpool School of Tropical Medicine, Liverpool, United Kingdom

## Abstract

Gerry Killeen and colleagues argue that malaria eradication efforts will not be successful until a better understanding of the ecology and evolution of the mosquito vectors is gained.

**Summary Points**

- Existing front-line vector control measures, such as insecticide-treated nets and residual sprays, cannot break the transmission cycle of *Plasmodium falciparum* in the most intensely endemic parts of Africa and the Pacific
- The goal of malaria eradication will require urgent strategic investment into understanding the ecology and evolution of the mosquito vectors that transmit malaria
- Priority areas will include understanding aspects of the mosquito life cycle beyond the blood feeding processes which directly mediate malaria transmission
- Global commitment to malaria eradication necessitates a corresponding long-term commitment to vector ecology

## Introduction

The Global Malaria Eradication Program, launched in the middle of the last century, over-promised and under-delivered [1]. Decades of pessimism followed, during which malariologists were afraid to even mention the goal of this program by name [2]. The term *eradication* was often nervously referred to as “the E-word” by a disillusioned community that had learned from bitter experience that optimistic forecasts [3] had been based on an oversimplified view of transmission ecology [4]. Eradication of malaria remains beyond our grasp today, but is nevertheless firmly back on the global health agenda as a long-term target [5].

## Ecological Obstacles to Eradication with Existing Interventions

By definition, eradication of human malaria parasites globally [5] requires that intervention options are available that can eliminate transmission anywhere in the world. Leading vector control technologies such as insecticide-treated nets (ITNs) and indoor residual spraying (IRS) can suppress transmission by one or even two orders of magnitude [4],[6] and dramatically alleviate disease burden [7],[8]. Nevertheless, these measures alone are not sufficient to eliminate transmission in large tracts of tropical Africa where the entomological inoculation rate (EIR), the most direct measure of human exposure, can exceed a thousand infectious bites per person per year [9],[10]. Expressed in terms of the parasite's reproductive number, this means that if the local parasite population were entirely eliminated by mass drug administration, for example, a single infected person moving into the area could give rise to as many as ten thousand new infections and readily re-establish stable transmission [10]. Under such conditions, simulations predict that even 100% coverage of an entire population with ITNs exhibiting near-ideal properties will fail to push the EIR below the threshold required for local elimination [11]. Although massive benefits of increasing ITN and IRS coverage have been achieved in many parts of equatorial Africa, elimination has remained elusive except for regions on the edge of stable transmission in Kenya, Tanzania and The Gambia (e.g. [12]–[14]). Evidence from the previous malaria eradication drive [4],[15] and contemporary initiatives [8],[16],[17] indicate that transmission remains robust in areas where it has been historically high. We argue here that a failure to appreciate the biological complexities that allow vector populations to resist or evade interventions has substantially impeded control efforts. In particular, we identify seven ecologically imposed obstacles that have limited the effectiveness of vector control, and must be tackled in order to move from control to eradication (Box 1).

**Box 1. Ecological Obstacles to Vector Control**

**(1) Variation in mosquito behaviour**

All front-line vector control methods used in Africa today (e.g., ITNs, IRS) are based on the stereotyped view that vectors bite and rest primarily inside houses. This assumption is based on the early characterization of *Anopheles gambiae* and *An. funestus* behaviours of feeding and resting almost exclusively indoors [49]. However, even these endophilic species feed outside to some degree, and may do so increasingly in response to domestic interventions [21],[22]. Crucially, many other primary vectors do not conform to this traditional model and often bite outdoors [49] (e.g., *An. arabiensis,* which dominates transmission in much of Africa [50]). Variation in feeding behaviour within vector species may have a genetic basis [51], which raises the possibility that vector control measures could select for genotypes which are least likely to encounter the intervention. Even when vectors are highly endophilic [6],[29], the application of insecticides in and around houses has fundamental limitations, because exhaustive coverage of all resting sites with IRS [22], or all humans with ITNs [6] is not possible in practice.

**(2) Insecticide resistance**

The ability of vectors to evolve diverse resistance mechanisms to insecticides has been well documented [20]. Resistance to all major classes of insecticides used against malaria vectors has now been recorded in Africa [52]. Recent evidence from dengue mosquito vectors indicates that permethrin resistance can increase by more than 100-fold in vector populations within just 7–8 y [53]. The capacity of vectors to develop resistance so rapidly will undoubtedly pose a major obstacle to malaria control based exclusively on insecticides.

**(3) Behavioural avoidance**

The emergence of new vector behavioural phenotypes is a less-recognized phenomenon than insecticide resistance, but it has the potential to similarly diminish the effectiveness of current interventions. Documented examples of adaptable vector behaviours that could impact interventions such as ITNs and IRS include changes in host-species preferences [27], and feeding outdoors or in the early evening when people are not protected by their houses or bed nets [21],[22]. During the last malaria eradication drive, several accounts of mosquitoes shifting from feeding inside to outside, and from humans to animals, were reported in response to insecticide use indoors [54]. Whether these behavioural shifts were a consequence of phenotypic plasticity or evolutionary change within vector populations is unknown. Regardless of the mechanism, such behavioural plasticity limits contact between vectors and insecticides, thus diminishing the effectiveness of the interventions that use them [21].

**(4) Vector biodiversity**

Over 30 different primary vectors dominate transmission in various parts of the world [55]. Many of these are part of species complexes and are represented by several genetically distinct chromosomal and molecular forms within a species that have distinct ecological and behavioural niches [51]. In addition to this complexity within primary vectors, low levels of transmission are frequently maintained by a myriad of behaviourally and ecologically diverse secondary vectors. Although these species are routinely ignored, even the small fraction of transmission they generate may be sufficient to sustain *Plasmodium* spp. in human populations [56]. The diversity of vector species increases outside of Africa, and presents a huge challenge to conventional methods of vector control [57]. Furthermore, vectors currently viewed as “secondary” may expand to dominate residual transmission and act as *de facto* primary vectors following the successful implementation of interventions aimed at current priority vector species [58].

**(5) Competitive and food web interactions**

Mosquito vectors are embedded within ecological communities where they act as predators, prey, and competitors. Consequently the reduction of one target vector may trigger a cascade of ecological effects that could impede or enhance transmission by another. Studies have reported that suppression of one vector species through habitat change or control was followed by an increase in another. Notable examples include the apparent replacement of *An. funestus* by *An. rivolurum* [59], and *An. parensis* [60] in areas of east Africa following house spraying. These changes were attributed to a reduction in interspecific competition caused by the intervention that allowed these secondary vectors to move into the niche formerly occupied by *An. funestus*.

Virtually nothing is known about the role of vectors in regulating, or being regulated by, their prey or predators. Consequently the potential use of biological control to manage vector populations is vastly underexplored. Perhaps the best-described biological regulator of vector populations is themselves. Several studies have shown that traits such as the larval development, fecundity, survival, and population growth rates of mosquito vectors is negatively correlated with their population size [61]–[63]. This density dependence means that as vector populations fall in response to interventions, the individual vectorial capacity of the remaining survivors may be significantly greater than that of the average mosquito pre-intervention. Consequently vector populations may become increasingly difficult to suppress as their abundance moves towards zero. Complementary approaches may therefore be required to eliminate residual transmission by vector populations which have been reduced far below their carrying capacity by interventions.

**(6) Dispersal and mating behaviour**

Knowledge of mosquito dispersal range is essential for accurate predictions of the optimal spatial implementation of more conventional control methods such as ITNs, IRS, and larviciding [64]–[66], and of the rate of spread of resistance genes. Unfortunately, direct observations of the dispersal ability of malaria vectors have been made in only a limited subset of vector species, environments, and experimental conditions, and few generalities can be made for this behaviour. Additionally, the control of vector populations and/or their disease transmission ability through the release of genetically modified or sterile males will depend on both the dispersal ability of released individuals and their ability to successfully compete for wild females. Efforts to ensure the reproductive success of such males are hampered by large knowledge gaps in our understanding of the environmental, genetic, and phenotypic determinants of male mating competitiveness, survival, and dispersal ability under natural conditions [30].

**(7) Environmental change**

Climate and environmental change are driving the expansion of numerous vector species and the intensification of pathogen transmission in many locations [67]. Specific examples include deforestation, which has prompted an increase in the human-biting rate of formerly zoophilic vectors in several parts of the tropics and the instigation of new malaria epidemics [68],[69]. Historical and forecasted rises in temperature have also been implicated in the spread of malaria into new habitats and regions [70]. Mitigating against the detrimental impacts of environmental change on malaria transmission will be particularly difficult when public health goals conflict with economic development. For example, following the elimination of malaria in the Demerara River Estuary of Guiana (by DDT spraying), the human population grew rapidly and land use activities switched from livestock herding to more profitable rice farming [25]. The removal of livestock from the landscape, however, caused the formerly zoophilic *An. aquasalis* to switch its feeding from livestock to humans. This change initiated the return of transmission into the area after 16 years of absence [25]. Irrigation and dam construction have also been linked to an increase in malaria risk, although the nature of the effect varies substantially between epidemiological, entomological, and socioeconomic settings [71]. While environmental changes to enable poverty reduction are essential to economic development and infectious disease control, sustaining malaria eradication will require a clearer mechanistic understanding of the impacts of both vector control and concurrent changes in natural resource management and land use activities.

## Thinking Outside the House

**Figure 1:**
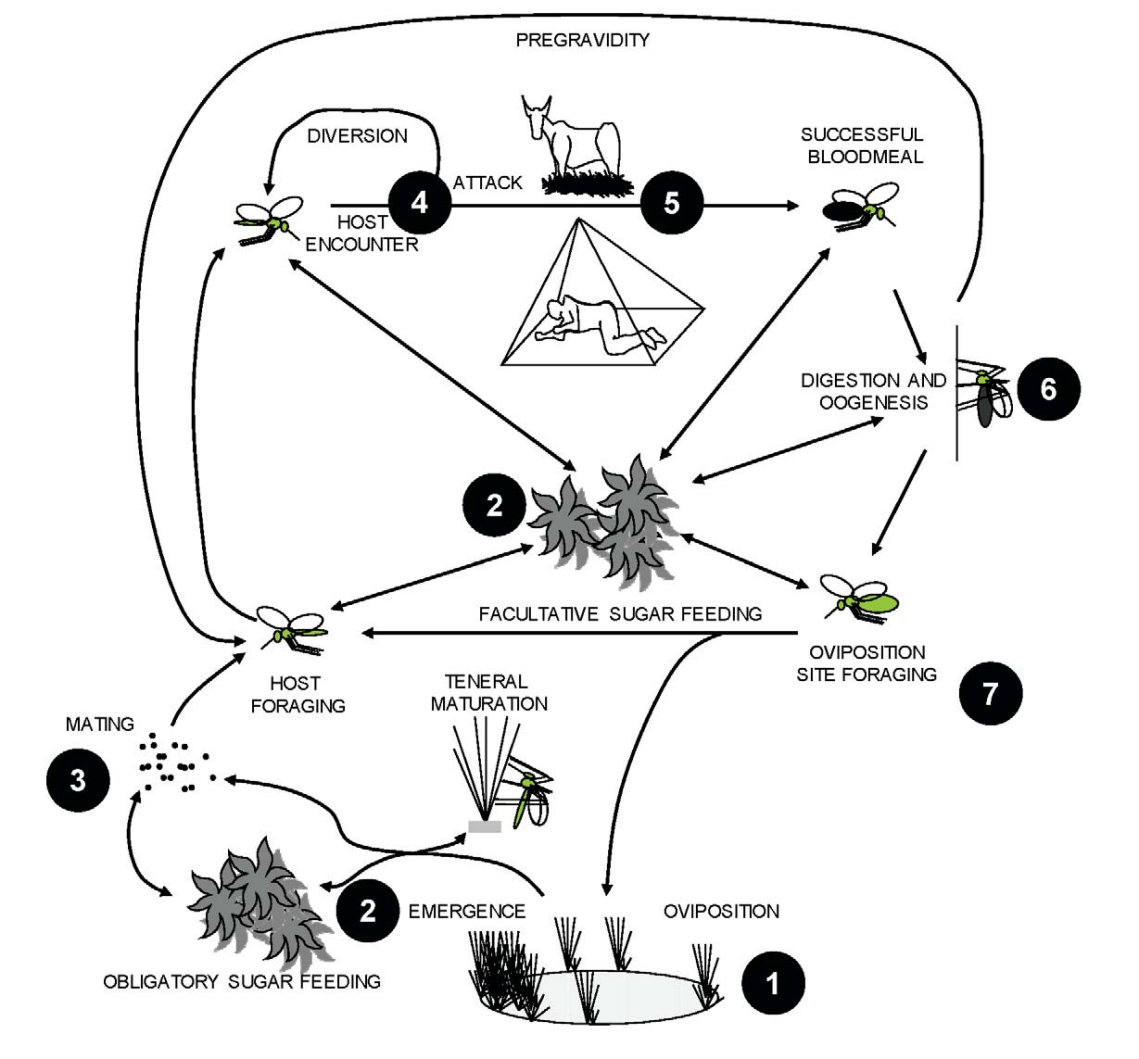
Life cycle components of malaria vector mosquitoes and corresponding examples of targets for novel intervention strategies. (**1**) Environmental management [39] and larvicide application by direct means [32],[33] or by autodissemination via adults [37]; (**2**) insecticide application to natural sugar sources [35], toxic sugar baits [36], and paratransgenic bacteria [40]; (**3**) pheromone trapping [41] and release of genetically modified or sterile males [30],[42]; (**4**) spatial and contact repellents [43] that work both indoors and outdoors and physical barriers to prevent mosquito entry into houses [44]; (**5**) zooprophylaxis [29], insecticide-treated cattle [45], and odour-baited traps [46]; (**6**) adult contamination with biological [47] and chemical [37] agents which may be autodisseminated; and (**7**) environmental management of water resources for adult vector control through increased mortality cost of foraging for oviposition sites [48].

The ecological hurdles detailed in Box 1 imply that there exists a fundamental limit to the degree of control that can be achieved with ITNs or IRS. In most settings, achieving elimination will require interventions which target mosquitoes outside of human habitations. Existing and new interventions must be combined into integrated packages [18],[19] that control mosquitoes at multiple points in the continuum from egg to adult, by targeting the key environmental resources upon which they rely to complete their life cycle: aquatic larval habitat, mates, sugar sources, blood hosts, and resting sites (Figure 1). With the exception of blood, very little is known about how mosquitoes use these resources or how to manipulate them so that malaria transmission is interrupted. We conclude that a better understanding of all aspects of vector ecology will inevitably yield numerous new and mutually complementary targets for integrated vector control. Ecology is therefore a prerequisite to eradication or elimination, and will be essential to sustaining success in the long term.

Historically, vector biologists have focused primarily on evaluating specific control interventions and less on fundamental studies of vector ecology. Now that the gap between currently achievable levels of control and the ultimate goal of eradication is becoming clear, new intervention options for integrated vector management [18],[19] are urgently needed. Strategic investment in vector ecology will thus be an essential enabling step towards malaria eradication. The ways in which vectors utilize resources vary from one environmental setting to another, so a clear understanding of such ecological processes is essential for identifying intervention strategies which work within a range of settings. Such demonstration of ecological generalisability, as well as scalability in the context of available human resource capacities, will be essential for ensuring the success of developing country control programs. Furthermore, the long-term effectiveness of any control strategy will depend on whether vectors respond to the evolutionary selection pressure created by interventions. For example, mosquitoes may respond by phenotypic plasticity, or by evolving traits such as insecticide resistance [20] or behavioural avoidance [21],[22]. Numerous examples of such phenotypic and genetic changes have been documented in response to previous control efforts (e.g., Box 1) and are likely to influence the sustainability of future eradication attempts. Consequently, understanding the likelihood of and rate at which such evolutionary changes can occur is vital for mitigating any detrimental epidemiological consequences they may bring. Finally, the applicability of any vector control strategy will depend on the dynamic human component of vector ecology, particularly the political, social, and economic factors that determine land and water use within afflicted communities. Knowledge of the quantitative relationship between these behaviours and malaria hazard, vulnerability, and risk is vital [23],[24]. Ultimately both human and vector populations will readjust their distribution and behaviour in response to changing patterns of ecological resources, sometimes with catastrophic effects for the maintenance of control efforts (e.g. [25]). While vector control cannot and should not come at the expense of impeding economic development which could promote a wider strengthening of health care and protective measures, all efforts should be made to identify and mitigate any potential conflicts between land use and vector control before they arise.

## So Where Do We Stand Right Now?

Our understanding of the ecology of mosquitoes that transmit malaria lags decades behind that of agricultural pests, endangered species, and model organisms. The reasons are multifaceted [26],[27], and disincentives include the lack of ecological representation and thus support on the funding panels of biomedical donors, limited training opportunities in fundamental ecology for medical entomologists, and the necessary ethical restrictions upon the types of experimental manipulations that are widely used to gain valuable insights into the population, community, and ecosystem dynamics of other insects [28] which do not transmit pathogens to humans. Examples of procedures which can yield crucial scientific information, but which are increasingly difficult to justify ethically include human landing catches [21] (because of the exposure risks they entail) and mark–recapture studies of mosquito demography and dispersal (because of community concerns about the re-release of potentially infectious mosquitoes that could instead have been killed). Evaluation of the potential use of alternative animal hosts to divert mosquitoes from biting humans also poses potential risks; theoretical simulations indicate there are plausible scenarios under which this may increase transmission by increasing blood availability and vector survival [29].

The paucity of national funding schemes for ecological research within impoverished malarious countries, and limited access to relevant overseas funding, have restricted the conversion of indigenous talent into an adequate expertise base. Furthermore, the primary focus of malaria control on developing countries with limited infrastructure or research capacity may deter the engagement of ecologists from the developed world who have a myriad of more convenient, accessible, and tractable organisms at their disposal. Until the inherent challenges of working in these more demanding settings is recognized and valued by mainstream ecology, researchers may have little incentive to build their careers in this area. Sponsors of fundamental biological research have typically undersupported vector ecology, on the basis of the assumption that public health and medical donors with often substantially larger budgets will fill this gap. Unfortunately this has rarely been the case in practice because donors generally prioritize applied research focusing on the development and delivery of interventions which have more obvious and immediate potential benefits [26].

Most funding for ecological studies of malaria vectors in recent years has been driven by the needs of specific biotechnological interventions [30],[31] rather than by the pursuit of basic ecological knowledge [26]. While this emphasis on applied research is clearly justified and understandable, benefits accrued will be short-lived unless such funding is matched by investment in the fundamental science that will provide new solutions to deal with resistance to current interventions and go beyond currently achievable levels of control to bring elimination realistically within reach.

As a result of these various funding deficiencies, huge knowledge gaps exist in relation to most components of the mosquito life cycle that occur outside of houses, including larval growth and sugar feeding, oviposition, and adult dispersal (Figure 1) [26],[31]. Even the development of delivery systems for the historically successful and recently rejuvenated strategy of physically eliminating or applying insecticides to larval habitats [32],[33] is severely limited by a paucity of suitable field survey methods and large-scale studies of aquatic-stage ecology [34]. An excellent example of what is possible with solid ecological observation and a little imagination comes from the deserts of Israel where dramatic reductions of malaria vector density around oases and cisterns, which may be comparable with dry-season refugia in Africa, were achieved using low-technology toxic sugar baits which are as lethal to mosquitoes as contact with an ITN [35],[36]. Similarly, results of a recent study of the effectiveness of exploiting resting and oviposition behaviours in *Aedes aegypti*, the primary vector of Dengue, to distribute insecticides to their own larval habitats [37] are encouraging. Nevertheless, the fact that no field estimates were available for any of the parameters of the coverage amplification model used to explain this success [37] highlights the knowledge gaps that may impede the use of this method against malaria vectors. Although further research into the behaviours that predispose vectors to such novel interventions is obviously attractive, a more integrated and holistic approach [26] is also required to maximize the value of ecological research as a means to identify additional strategies for controlling, eliminating, and eradicating malaria transmission.

## Making It Happen

The overarching strategic priority for increased investment should therefore be to improve the quantitative understanding of mosquito life history, fitness, genetics, and behavioural processes as determinants of their population stability and malaria transmission intensity. Support should be directed towards delivering key outcomes, without which malaria eradication is difficult to envisage (Box 2). As such, ecology should—like other basic disciplines such as molecular biology and bioinformatics—be considered an enabling science essential for defining the target product profiles of completely new control technologies and delivery systems. To achieve these outcomes and make malaria eradication a realistic ambition, we propose key areas for specific strategic investment (Box 3).

**Box 2. Key Outcomes of Enhanced Investment in Malaria Vector and Transmission Ecology**

(1) Identification of specific vulnerabilities of vector and sporogonic-stage parasite populations that can be prioritized for intervention development and delivery

(2) Estimation of threshold values of vector population parameters required to achieve pathogen elimination

(3) Quantification of the strength of selection pressures imposed on vectors by particular interventions so that the likelihood and rate at which physiological and behavioural resistance traits emerge can be predicted and managed

(4) Avoidance of the mistakes of the previous eradication drive through biologically realistic understanding of the scale of the challenge

(5) Estimation of achievable endpoints for single and multiple interventions and synergies and redundancies associated with particular combinations

(6) Stimulation of creative, “blue skies” scientific investigation resulting in identification of unforeseen novel intervention targets and strategies

**Box 3. Key Areas for Specific Strategic Investment in Ecological Research to Enable Malaria Eradication**

(1) Development of new field measurement tools for surveying diverse primary and secondary vector populations and the environmental conditions and resources they rely upon through all phases of their life cycles

(2) Establishment of comprehensive, long-term data collection systems spanning individual to landscape scales from diverse and representative field sites

(3) Creation and maintenance of public data repositories with standardized, simplified data storage formats for mosquito ecology data combined with policies and incentive systems that facilitate data sharing and synergy between laboratory- and field-based investigators

(4) Application of cutting-edge mathematical modelling approaches to understand vector populations dynamics, pathogen transmission, and optimal intervention strategies

(5) Development and application of enclosed, pathogen-free, semi-field mesocosms in which vector populations can be experimentally manipulated [38]

6) Exploitation of the perturbations of vector populations and parasite transmission processes resulting from ongoing scale-up of existing intervention measures so that the population dynamics, behavioural specialization, and competitive relationships between mosquito species can be lucidly understood

(7) Engagement and recruitment of leading theoretical and empirical ecologists into malaria vector research, control, and capacity strengthening

Direct research investment will be required to develop new field measurement tools, establish a network of longitudinal population monitoring sites with complementary semi-field facilities [38], apply advanced modelling approaches, and promote career and skills development of endemic-country scientists in both public health and vector ecology. Beyond these obvious needs, additional incentives are required to engage expert ecologists from more knowledge-rich fields into malaria vector ecology. While ecology-oriented funding agencies have a vital role in facilitating the reinvigoration of vector biology, the bulk of the required financing will ultimately have to come from the health sector funders and policy makers who have prioritized malaria eradication and committed themselves to the long, hard road towards this worthy but distant goal.

## Abbreviations

EIR: entomological inoculation rate
IRS: indoor residual spraying
ITN: insecticide-treated net
